# Impulsive Rats Exhibit Blunted Dopamine Release Dynamics during a Delay Discounting Task Independent of Cocaine History

**DOI:** 10.1523/ENEURO.0119-17.2017

**Published:** 2017-04-24

**Authors:** Travis M. Moschak, Regina M. Carelli

**Affiliations:** Department of Psychology and Neuroscience, University of North Carolina, Chapel Hill, NC 27599.

**Keywords:** behavior, cocaine, delay discounting, dopamine, impulsivity, reward

## Abstract

The inability to wait for a large, delayed reward when faced with a small, immediate one, known as delay discounting, has been implicated in a number of disorders including substance abuse. Individual differences in impulsivity on the delay discounting task are reflected in differences in neural function, including in the nucleus accumbens (NAc) core. We examined the role of a history of cocaine self-administration, as well as individual differences in impulsivity, on rapid dopamine (DA) release dynamics in the NAc core. Rats with a history of cocaine or water/saline self-administration were tested on delay discounting while being simultaneously assayed for rapid DA release using electrochemical methods. In controls, we found that cue DA release was modulated by reward delay and magnitude, consistent with prior reports. A history of cocaine had no effect on either delay discounting or DA release dynamics. Nonetheless, independent of drug history, individual differences in impulsivity were related to DA release in the NAc core. First, high impulsive animals exhibited dampened cue DA release during the delay discounting task. Second, reward delay and magnitude in high impulsive animals failed to robustly modulate changes in cue DA release. Importantly, these two DAergic mechanisms were uncorrelated with each other and, together, accounted for a high degree of variance in impulsive behavior. Collectively, these findings demonstrate two distinct mechanisms by which rapid DA signaling may influence impulsivity, and illustrate the importance of NAc core DA release dynamics in impulsive behavior.

## Significance Statement

Delay discounting, a form of impulsivity, reflects the inability to delay gratification and has been implicated in substance use disorders. Here, we determined the role of both cocaine experience and impulsivity on dopamine (DA) release dynamics in the nucleus accumbens (NAc) core during a delay discounting task. We found that reward delay and magnitude modulated cue DA release, but this was unaffected by a history of cocaine experience. However, independent of drug history, high impulsive animals exhibited dampened cue DA release throughout delays. Our findings demonstrate two distinct mechanisms by which NAc core DA may influence impulsivity and illustrate the importance of rapid DA signaling in the NAc core in impulsive behavior.

## Introduction

Delay discounting is a form of impulsivity critically involved in the maintenance of drug abstinence (Krishnan-Sarin et al., 2007; Washio et al., 2011; Stanger et al., 2012; see Pattij and De Vries, 2013). Individuals with a history of drug use, including cocaine, have heightened delay discounting (i.e., more impulsivity; Kirby et al., 1999; Mitchell, 1999; Coffey et al., 2003; Bjork et al., 2004; Hoffman et al., 2006; Bickel et al., 2014). Preclinical studies investigating the relationship between cocaine and delay discounting have shown that drug-naïve rodents with higher delay discounting acquire drug self-administration faster (Anker et al., 2009a) and have greater reinstatement for the drug after a period of abstinence (Broos et al., 2012). Furthermore, several studies have shown that repeated administration of cocaine increases delay discounting behavior in rats (Roesch et al., 2007b; Simon et al., 2007; Mendez et al., 2010; Hernandez et al., 2014; Mitchell et al., 2014), although these findings have not been consistently replicated (Winstanley et al., 2007; Broos et al., 2012; Xie et al., 2012).

Research in human subjects has shown that there is considerable overlap in the neural circuitry involved in impulsivity and drug abuse, particularly in frontal and limbic brain structures (for reviews, see Crews and Boettiger, 2009; Dalley et al., 2011). These studies highlight an important role of individual differences in brain structure and/or activity that correlate with individual disparities in both drug craving (Brody et al., 2007; Heinz et al., 2014) and impulsivity (Hariri et al., 2006; Ballard et al., 2015; Joutsa et al., 2015). Such findings extend to preclinical research, where individual aspects of neural function correlate with differences in drug sensitization, conditioned place preference, and drug self-administration (Striplin and Kalivas, 1992; Harris et al., 2007; Wheeler et al., 2008), as well as with differences in delay discounting behavior (Zeeb et al., 2010; Moschak and Mitchell, 2014; Pattij et al., 2014).

The mesolimbic dopamine (DA) system, particularly the nucleus accumbens (NAc) and its DAergic input, is critically involved in delay discounting behavior. For example, using electrochemical methods, our laboratory has shown cue-related DA signaling in the NAc core that tracks the delay and magnitude of rewards earned in a delay discounting task (Saddoris et al., 2015). Further, optogenetic activation of ventral tegmental area (VTA) DA terminals in the NAc during cues alters delay sensitivity (Saddoris et al., 2015). Other studies showed that lesions of the NAc core decrease choice of the large, delayed reward (Cardinal et al., 2001; Bezzina et al., 2007; Galtress and Kirkpatrick, 2010). Critically, individual differences play a role in this behavior, as inactivation of the NAc core only alters delay discounting in low impulsive animals (Moschak and Mitchell, 2014). Further, both systemic administration of amphetamine and *in vitro* electrical stimulation of NAc elicit less DA release in high impulsive discounters when compared with low impulsive discounters (Diergaarde et al., 2008; Zeeb et al., 2016).

It is unknown, however, what effect a history of cocaine self-administration has on DA release dynamics in the NAc core during delay discounting, or whether DA release during delay discounting varies depending on individual differences in impulsivity. Here, we examined rapid DA signaling during a delay discounting task, and determined whether a history of cocaine self-administration (2 h/d for 14 d) alters DA release dynamics and behavior. Next, we investigated the role of individual differences in impulsivity on DA release in animals with high and low impulsivity. Consistent with prior studies, we found that cue DA release in the NAc core was modulated by reward magnitude and delay, however, no aspect of this signaling or behavior was affected by a history of cocaine. Nevertheless, we found that independent of drug history, two discrete patterns of cue-induced DA release in the core independently tracked individual differences in impulsivity, as high impulsive rats exhibited dampened cue DA release throughout delays.

## Materials and Methods

### Subjects

Male Long Evans rats (*n* = 14; Charles River) initially aged 8–10 weeks and weighing 300–325 g were used. Animals were housed under a 12/12 h light/dark cycle (lights on at 7 P.M.) and were food restricted to no <85% free feed weight, receiving 7–10 g of 2020X Teklad food pellets/d (Harlan Laboratories). Experiments were conducted in accordance with the National Institutes of Health Guidelines for the Care and Use of Laboratory Animals and the University of North Carolina at Chapel Hill Institutional Animal Care and Use Committee.

### Apparatus

Voltammetric recordings during delay discounting were completed in custom-built operant chambers (43 × 43 × 53 cm) housed in a copper mesh Faraday cage sound-attenuated cubicle. Each chamber contained a houselight, white noise generator, two retractable levers with two cue lights above them, and a food receptacle equal distance between the levers. Self-administration sessions were conducted in separate, contextually distinct, operant chambers (25 × 25 × 30 cm) housed in sound-attenuating cubicles (Med Associates). One wall of these chambers contained a nosepoke device, a houselight, and a tone generator. The opposite wall contained two retractable levers with cue lights above them, and a water receptacle positioned between the levers. Cocaine/saline infusion was delivered via tubing running through a counterweighted arm and controlled by a motorized syringe pump. For both sets of chambers, behavioral events were recorded and controlled by a computer running Med-PC software (Med Associates).

### Surgical procedures

Animals underwent two surgeries for catheter implantation and electrochemistry. In both cases, animals were anesthetized with a mixture of 100-mg/kg ketamine hydrochloride and 10-mg/kg xylazine. In the first surgery, a catheter (Access Technologies) was implanted into the jugular vein, using procedures described previously (Carelli et al., 2000; Saddoris et al., 2016a,b). In a second surgery, conducted approximately five weeks later, rats were surgically prepared for voltammetric recording using standard laboratory procedures (Day et al., 2010, Sugam et al., 2012, Saddoris et al., 2015). Briefly, a guide cannula (Bioanalytical Systems) was positioned dorsally to the NAc core (anteroposterior, AP +1.3 mm, mediolateral, ML −1.3 mm from bregma) and another guide cannula (for the Ag/AgCl reference electrode) was placed contralateral to the NAc cannula. A bipolar stimulating electrode was placed dorsally to the VTA (AP −5.2 mm, ML −1.0 mm and dorsoventral, DV −7.0 mm from bregma) and ipsilateral to the NAc cannula. Correct placement of the stimulating electrode in the VTA was determined by applying a range of stimulation parameters (12–24 biphasic pulses, 20–60 Hz) and observing tail movement. The stimulating electrode was lowered in increments of 0.1 mm until slight to no tail movement was observed at 60 Hz, 24 pulses. Stainless steel screws and dental cement were then used to secure all items.

### Behavior

#### Delay discounting task

**Figure 1.**
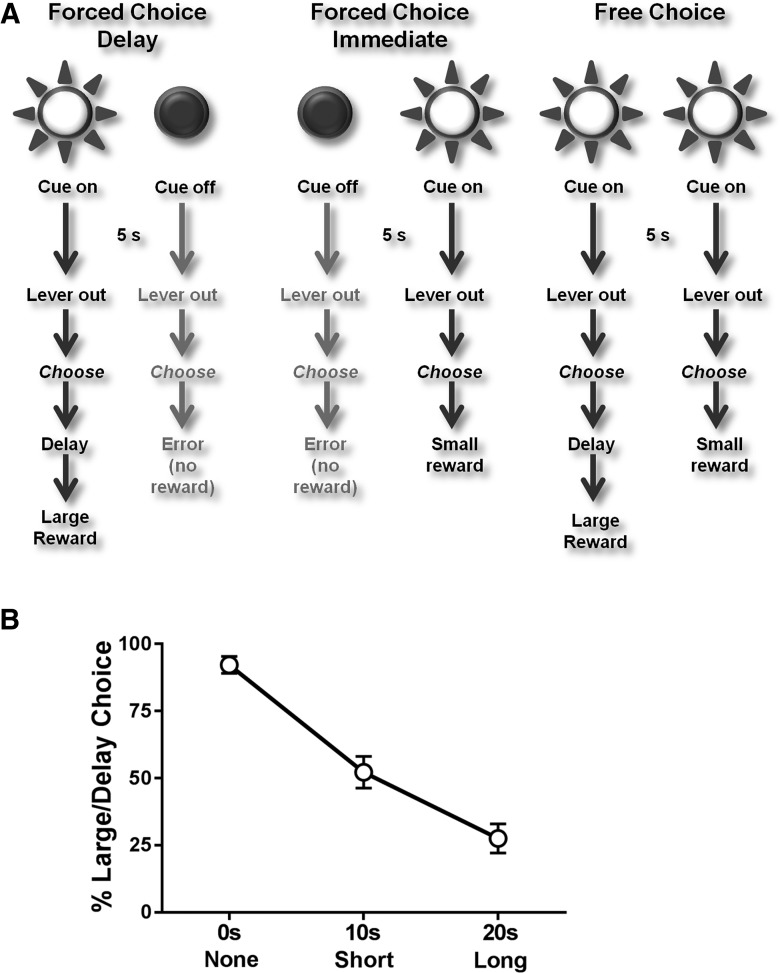
Delay discounting task and behavior. ***A***, Task schematic (see text for details). ***B***, Percentage choice of the large/delayed reward as a function of delay for control rats (average of the five test days). Rats decreased their percentage choice of the large reward as the delay to the large reward increased.

The delay discounting task was identical to that previously used in our lab (Saddoris et al., 2015). Animals were initially trained to press two levers for a sucrose pellet (45 mg, Purina TestDiet). Once animals had achieved 50 presses on each lever for two consecutive days, they began the delay discounting task. Each delay discounting session consisted of three blocks of 30 discrete trials (Fig. 1*A*). The first 20 trials of each block were mixed forced choice delay (Fig. 1*A*, left) and forced choice immediate (Fig. 1*A*, middle) trials (10 trials of each). For forced choice delay trials, a single cue light was illuminated over one lever for 5 s, followed by extension of both levers. Responses (FR1) on the lever below the illuminated cue light were rewarded with three sucrose pellets delivered after a delay [either no delay (block 1), short delay (10 s; block 2), or long delay (20 s; block 3)]. For forced immediate trials, the cue light above the second lever was illuminated for 5 s then both levers were extended into the chamber. Lever presses (FR1) under the illuminated cue light within 10 s were immediately rewarded with a single sucrose pellet across the three blocks. For both trial types, presses on the nonilluminated lever were not rewarded and counted as an error. Similarly, if the animal did not respond on either lever within 10 s, both levers retracted and the trial was counted as an omission. The next 10 trials within each block were free choice trials (Fig. 1*A*, right) in which both cue lights simultaneously illuminated for 5 s, and both levers were extended. Once either lever was pressed, both levers retracted and the animal was rewarded based on the contingency of reinforcement for the chosen lever within that block. Failure to choose a response within 10 s resulted in the levers retracting and the trial counted as an omission. Importantly, each trial was of fixed duration (60 s) so that reward choice was not influenced by how quickly the rat could complete the task, i.e., choosing the smaller immediate reward did not lead to the next trial more quickly.

#### Self-administration

Next, animals underwent intrajugular catheter implantation, and one week later, began self-administration training. Here, the nosepoke port was illuminated and if the animal made a nosepoke response, cocaine (0.33 mg in 0.2 ml of 0.9% saline, i.v.) or an equal volume of both saline (i.v.) and water (delivered to the reward receptacle) was delivered paired with a 30 s houselight-tone compound stimulus. Additional responses during this 30-s period were recorded but had no programmed consequences. Each session lasted for 2 h/d for 14 d. All rats were mildly water-restricted (∼30 ml/d) but given *ad libitum* food access. Cocaine hydrochloride was obtained from the National Institute on Drug Abuse.

### Fast-scan cyclic voltammetry (FSCV)

Following self-administration training, animals resumed *ad libitum* water access and were food-restricted to no <85% of free-feed weight. Approximately two weeks later, all animals underwent surgery for voltammetric recordings, described above. Following recovery, animals were given 2 d of delay discounting to reacclimate them to the task. They were then tested for five consecutive days of delay discounting (days 24–28 following cessation of self-administration). Rapid DA signaling was measured during the delay discounting task on one of the test days using FSCV, as previously described (Day et al., 2010, Sugam et al., 2012, Saddoris et al., 2015). Briefly, on the test day, a carbon-fiber microelectrode was lowered into the NAc core with a locally constructed microdrive (Chemistry Department Electronic Facility, University of North Carolina, Chapel Hill), after placing an Ag/AgCl reference electrode in the contralateral hemisphere. The carbon-fiber microelectrode was held at −0.4 V versus the Ag/AgCl reference electrode. Periodically a cyclic voltammogram was acquired (100-ms intervals) by applying a triangular wave form that drove the potential to 1.3 V and back to −0.4 V. Changes in current at the oxidation potential for DA were compared with electrically-stimulated DA release at the same location. Chemometric analysis was used to identify DA concentrations using HDCV software (UNC Chemistry Electronics) and aligned to behavioral events (Trans IV, MED Associates).

Analysis of FSCV recordings was similar to previous reports (Phillips et al., 2003; Stuber et al., 2005a,b; Owesson-White et al., 2009, Day et al., 2010, Sugam et al., 2012, Cameron et al., 2014, Saddoris et al., 2015). Briefly, each subject received electrical stimulation of the VTA (frequency: 12–60 Hz, pulses: 1–20) to generate a training set of DA release at the recording location in the NAc. To analyze recorded FSCV data, each subject’s training set collected from the site of recording was used to chemometrically convert recorded current during the session into DA concentration (Rodeberg et al., 2015). Concentrations were then aligned to behavioral events to assess DA release dynamics relative to task stimuli.

### Histology

On completion of the experiment, voltammetry animals were euthanized with an overdose of ketamine/xylazine. A 250-µA current was passed through a tungsten electrode for 5 s to create a lesion and the brains were subsequently extracted and stored in 20% sucrose/10% formalin. Brains were sectioned at 40 µm by a cryostat and mounted on slides to verify placement of the electrode according to an atlas (Paxinos and Watson, 1998). For cocaine and water/saline rats, electrode tips were located in the NAc core, from +1.2 to +2.0 relative to bregma.

### Data analysis

#### Behavior

To compare acquisition of self-administration between groups (cocaine vs water/saline controls), the number of nosepokes was analyzed via a mixed two-way ANOVA. Analysis of delay discounting behavior has been described previously (Saddoris et al., 2015). Briefly, following self-administration, a two-way ANOVA (delay × group) was used to compare percentage choice of the large reward between the two groups, averaged across the five test days. Additional three-way ANOVAs (delay × magnitude × group) were calculated to compare errors, omissions, and latency to press across trial types.

#### FSCV

Peak DA was assessed for each trial by determining the maximum concentration in the 2-s time window following cue onset. A three-way mixed ANOVA determined changes in peak DA as a function of reward delay, reward magnitude, and drug history. Cumulative DA was determined by summing the DA concentration of each of the 100-ms time bins for the 5 s following cue onset to give an area under the curve. A three-way mixed ANOVA was calculated to determine changes in cumulative DA as a function of reward delay, reward magnitude, and drug history. To determine whether DA encoded the relative value of the large versus small reward cue as a function of delay, we subtracted the peak DA for the small reward cue from the peak DA for the large reward cue. These values were then correlated with percentage choice of the large reward for each delay.

Finally, we investigated the relationship of impulsivity to DA release dynamics and its possible interaction with drug history. Impulsivity was determined by averaging the percentage choice of the large reward across all three delays on the day voltammetry was measured, and animals were divided into high and low impulsivity groups via median split. As above, mixed three-way ANOVAs were run to measure the relationship of impulsivity to peak DA and cumulative DA. An additional two-way (delay × impulsivity) ANOVA was run on relative peak cue DA (peak DA for large reward cue – peak DA for small reward cue) in addition to a *t* test examining the differences between high and low impulsive rats in the change in relative peak cue DA from the 0- to 20-s delay. These analyses were then repeated with an additional factor (drug). Next, Pearson correlations were used to analyze the relationship between impulsivity and peak DA, cumulative DA, and change in relative peak cue DA. To further analyze these data at each individual delay block, we correlated the aforementioned DA variables with choice behavior in each delay block alone (0, 10, 20 s; relative peak DA was used rather than change in relative peak DA, since we could not calculate change scores within block). Finally, to determine the combined relationships between cumulative DA, change in relative peak cue DA, and impulsivity, we ran a linear regression model with cumulative cue DA and the change in relative peak cue DA as independent variables and impulsivity as the dependent variable. This analysis was repeated as above using each individual delay block (0, 10, 20 s). For all within-subjects analyses that did not pass Mauchly’s test for sphericity, Huynh-Feldt adjusted degrees of freedom were used.

## Results

### Self-administration behavior

Following surgery, rats were randomly subdivided into two groups and trained to nose-poke for either intravenous cocaine, or for intravenous saline paired with water delivered into the reward receptacle (controls). Both groups of rats completed similar numbers of nose-pokes for their respective rewards. Across the last 3 d of training, there were no significant differences in total number of nose-pokes between groups (cocaine: 25.14 ± 1.93 nose-pokes/d; water/saline: 26.24 ± 2.46 nose-pokes/d; *t*_(12)_ = 0.35, *p* = 0.732). Cocaine rats administered 19.02 ± 1.25 mg/kg/d of cocaine and water/saline rats administered 5.25 ± 0.49 ml/d of water/saline.

### Reward delay and magnitude modulate DA release dynamics to cues during delay discounting in control (water/saline) rats

Control rats exhibited classic delay discounting behavior, where their responses for the large reward declined as the delay to obtain it increased (Fig. 1*B*). During free choice trials, rats’ initial preference for the large-reward lever decreased as delays for that outcome increased across blocks (*F*_(2,12)_ = 73.92, *p* < 0.001). In the no-delay block, rats strongly preferred the large (immediate) option, choosing it significantly greater than chance (*t*_(6)_ = 13.25, *p* < 0.001). In the short-delay block (delay to the large reward increased to 10 s), preference for the large-delay lever decreased to a rate equal to the small-immediate option (*t*_(6)_ = 0.37, *p* = 0.721). In the long-delay block (large reward delay: 20 s), rats now strongly preferred the small-immediate option, selecting it well above chance (*t*_(6)_ = 4.10, *p* = 0.006).

**Figure 2.**
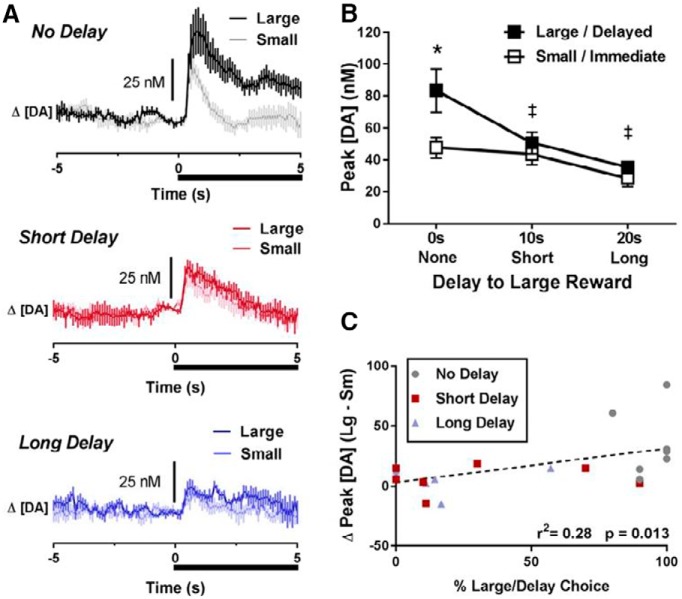
DA release dynamics relative to the cue for rats with a history of water/saline self-administration (controls). ***A***, Change in [DA] as a function of time for each cue during each delay block. Cue onset and duration is indicated by the black bar below each trace. ***B***, Changes in peak cue DA as a function of magnitude and delay. Peak DA was significantly greater following the large reward cue than the small reward cue at the 0-s delay. However, peak DA to the large reward cue significantly decreased at the 10- and 20-s delays. ***C***, The relationship between relative peak cue DA (peak cue DA for the large reward cue minus peak cue DA for the small reward cue) and subsequent choice behavior during free choice trials. Higher relative peak cue DA for the large reward predicted greater choice of the large reward. *p < 0.05 comparing large reward cue to smaller reward cue, ‡*p* < 0.05 comparing large reward cue at 0 s to large reward cue at 10 and 20 s.

We have previously shown that cues predictive of available choices during delay discounting behavior evoked DA release that scaled with the rat’s preferred choices and dynamically shifted as delay to reinforcement for the large reward increased (Saddoris et al., 2015). Here, we observed similar findings for our water/saline control rats. Across all control rats, cue DA release changed as a function of both magnitude and delay, as illustrated in Figure 2*A*,*B*. In Figure 2*A*, DA concentration is aligned to cue onset (black bar, time 0) on forced choice large versus forced choice small reward trials during the three delay blocks. Figure 2*B* shows peak DA concentration following cue onset for forced large/delayed cues (black squares) and forced small/immediate cues (open squares) across each reward delay block. A two-way ANOVA revealed a significant main effect of magnitude (*F*_(1,6)_ = 10.29, *p* = 0.018), a significant main effect of delay (*F*_(2,12)_ = 12.66, *p* = 0.001), as well as a significant interaction of magnitude and delay (*F*_(2,12)_ = 8.07, *p* = 0.006) on peak DA concentrations. That is, peak DA to the cue was largest for the large reward with no delay, and this relative difference subsequently declined as a function of delay. However, unlike our previous report (Saddoris et al., 2015), cue DA for the small reward cue was not larger than cue DA for the large reward cue at the longest (20 s) delay (*t*_(6)_ = 1.65, *p* = 0.15). Nonetheless, greater relative peak cue DA release (change in peak DA) to the large cue during forced choice trials predicted greater percentage choice of the large reward as a function of delay during free choice trials (*r* ^2^ = 0.28, *p* = 0.013; Fig. 2*C*), suggesting that NAc core DA tracked the more valuable option as well as future behavioral choice.

### A history of cocaine does not alter delay discounting behavior or DA release dynamics during delay discounting

**Figure 3.**
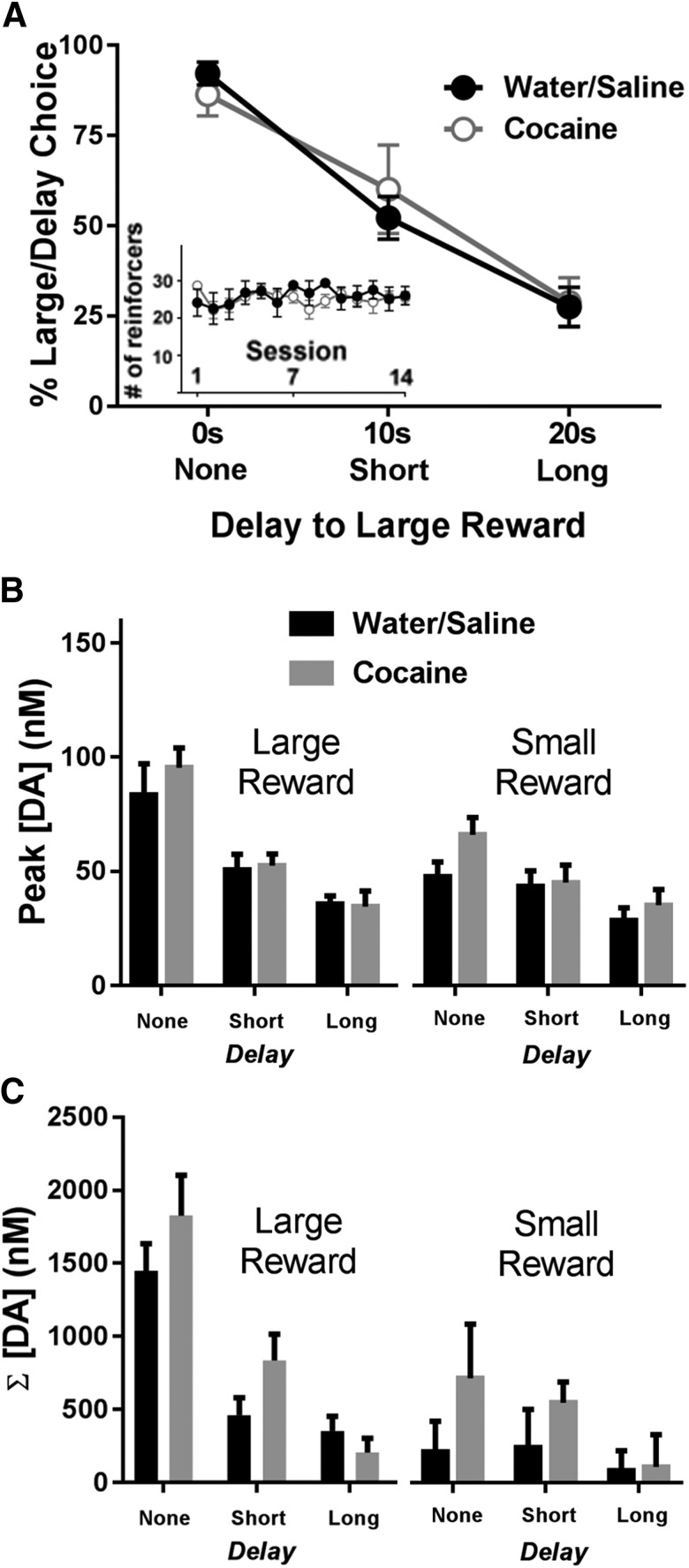
Effect of a history of cocaine on delay discounting and DA release dynamics. ***A***, Animals with a history of cocaine or saline self-administration did not differ in their percentage choice of the large, delayed reward during the delay discounting task (average of the last 5 d). Inset, Water/saline and cocaine rats earned similar numbers of reinforcers across the 14 self-administration sessions. ***B***, Differences in peak cue DA for each magnitude and delay across groups. There were no differences in peak cue DA between animals with a history of water/saline or cocaine self-administration. ***C***, There were no differences in cumulative DA during cues between animals with a history of water/saline or cocaine self-administration.

One objective of the present study was to determine whether a history of cocaine self-administration alters delay discounting behavior and simultaneously measured DA release dynamics within the delay discounting task. Figure 3*A* shows delay discounting curves for animals with a history of cocaine self-administration and controls. A two-way ANOVA revealed a main effect of delay to the large reward (*F*_(2,24)_ = 91.08, *p* < 0.001), but no effect of group (*F*_(1,12)_ = 0.17, *p* = 0.898) or interaction of group X delay (*F*_(2,24)_ = 1.14, *p* = 0.337). Additionally, there were no differences in latency, errors, or omissions between the two groups (all analysis of variance interactions, *p* > 0.05). Finally, there were no differences across the five test days, and degree of impulsivity in each rat did not significantly differ during voltammetry test days (all analysis of variance interactions, *p* > 0.05).

Despite a lack of behavioral differences, we also examined whether there were alterations in DA release dynamics following a history of cocaine. First, as shown in Figure 3*B*, we graphed peak DA concentrations for the large (left) versus small (right) reward as a function of delay. A three-way ANOVA revealed main effects of delay (*F*_(2,24)_ = 31.94, *p* < 0.001) and magnitude (*F*_(1,12)_ = 20.17, *p* = 0.001), but no main effect of group (*F*_(1,12)_ = 1.08, *p* = 0.319). There was a significant delay × magnitude interaction (*F*_(2,24)_ = 14.11, *p* < 0.001), but all other interactions were not significant (*p* > 0.05). Second, as shown in Figure 3*C*, there were no significant differences in cumulative DA across groups. Specifically, a three-way ANOVA revealed main effects of delay (*F*_(1,24)_ = 17.13, *p* < 0.001) and magnitude (*F*_(1,12)_ = 18.30, *p* = 0.001), but no main effect of group (*F*_(1,12)_ = 1.60, *p* = 0.231); other than a significant delay × magnitude interaction (*F*_(2,24)_ = 21.73, *p* < 0.001), all other interactions were not significant.

### DA release dynamics in impulsive rats are blunted during the task

**Figure 4.**
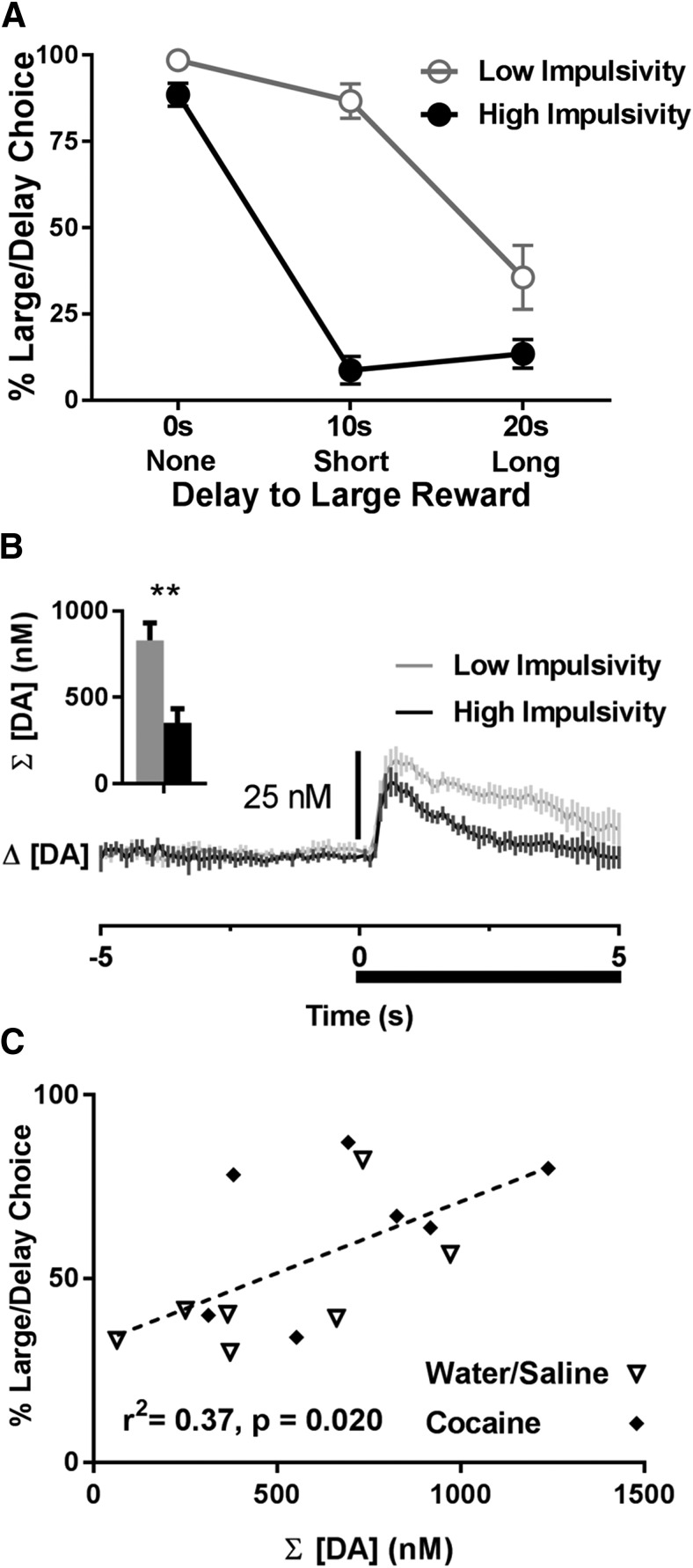
Differences in cue DA as a function of impulsivity. ***A***, Animals grouped as having low (history of cocaine, *n* = 5; water/saline, *n* = 2) or high (history of cocaine, *n* = 2; water/saline, *n* = 5) impulsivity exhibited significantly different delay discounting behavior. ***B***, Change in cue DA as a function of time (average of all delays and magnitudes). Cue onset and duration is indicated by the black bar below the trace. Inset, Animals with low impulsivity had significantly higher cumulative cue DA than animals with high impulsivity, **p < 0.01. ***C***, Percentage choice of the large/delayed reward significantly correlated with cumulative cue DA.

The above findings indicate that a history of cocaine self-administration did not alter delay discounting behavior or DA release dynamics in the NAc core. However, it was clear that level of impulsivity, a key feature of delay discounting behavior, precipitated differences in both behavior and DA signaling in the present study, independent of cocaine history. Here, rats were divided by median split into two distinct groups based on their level of impulsivity (low vs high) during the task, independent of drug history. A two-way ANOVA (delay × impulsivity group) revealed a main effect of delay (*F*_(2,24)_ = 101.12, *p* < 0.001), a main effect of impulsivity group (*F*_(1,12)_ = 65.49, *p* < 0.001, and a significant delay by impulsivity group interaction (*F*_(2,24)_ = 26.88, *p* < 0.001), demonstrating that the high impulsive rats shifted to the small, immediate reward sooner compared with low impulsive rats (Fig. 4*A*). Importantly, high impulsive and low impulsive rats did not differ on number of omissions, errors, or latencies to respond (all analyses *p* > 0.05).

Next, we examined whether differences in impulsive behavior were accompanied by differences in cue DA release dynamics (Fig. 4*B*). First, we examined peak DA concentrations across groups during the task. A three-way ANOVA showed main effects of delay (*F*_(2,24)_ = 35.52, *p* < 0.001) and magnitude (*F*_(1,12)_ = 21.21, *p* = 0.001), but no main effect of impulsivity group (*F*_(1,12)_ = 0.99, *p* = 0.339) and no significant interactions with impulsivity (all *p* > 0.05) on peak DA. In contrast, cumulative DA release dynamics differed across the high and low impulsivity groups during the task. A three-way ANOVA revealed main effects of delay (*F*_(2,24)_ = 17.48, *p* < 0.001), magnitude (*F*_(1,12)_ = 18.31, *p* = 0.001), and impulsivity group (*F*_(1,12)_ = 13.30, *p* = 0.003) on cumulative cue DA (all interactions with impulsivity group were not significant). As illustrated in Figure 4*B*, inset, high impulsive animals had significantly lower cumulative DA during cues, collapsed across delays and magnitudes, than their low impulsive counterparts. To further assess these differences, we ran a correlation between percentage choice of the large, delayed lever and cumulative cue DA, as shown in Figure 4*C*. We found that high cumulative DA was significantly correlated with low impulsivity (i.e., high percentage choice of the large, delayed reward; *r* ^2^ = 0.37, *p* = 0.020); this finding was not significant when using data only from each individual delay (0 s alone, 10 s alone, and 20 s alone; *p* > 0.05). To ensure that this finding was not related to drug history, we examined the same parameters with drug history as a factor; critically, there were no significant interactions between impulsivity and drug history (all analysis of variance interactions, *p* > 0.05).

**Figure 5.**
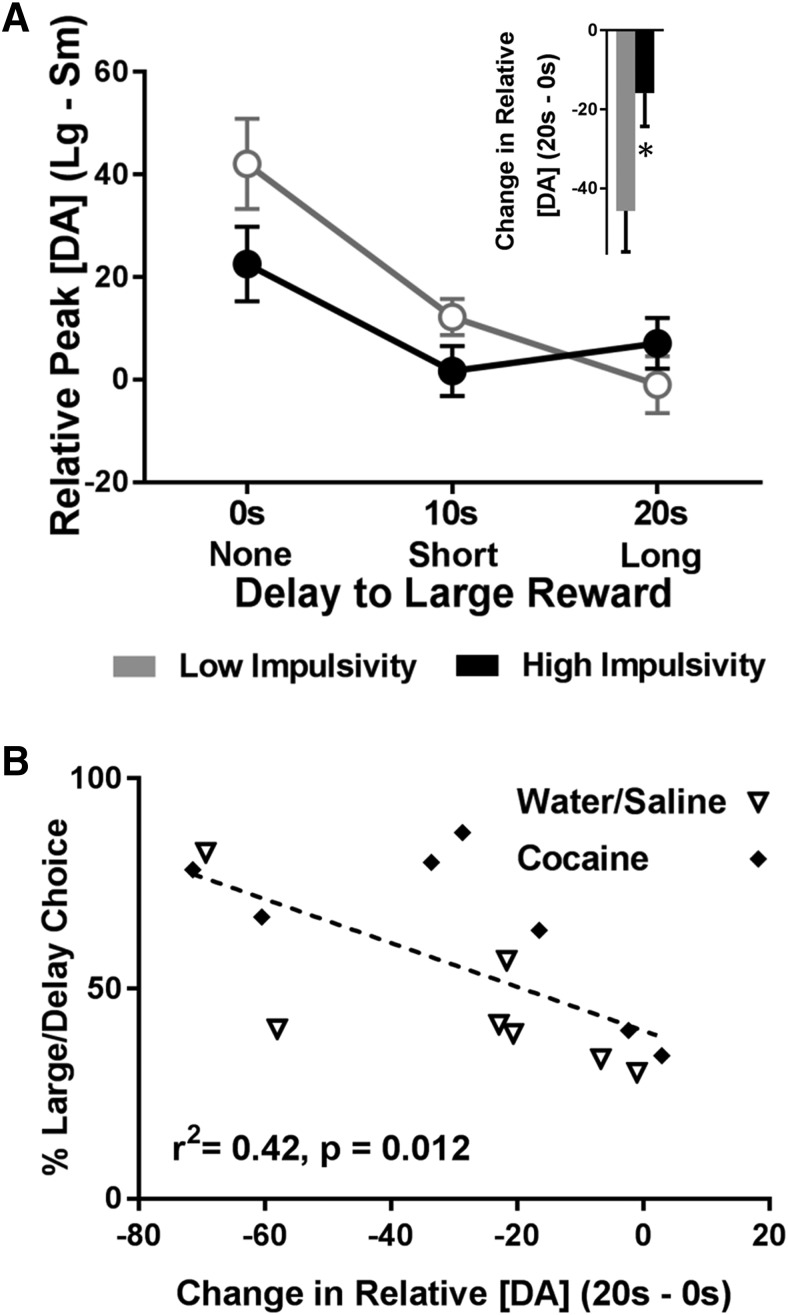
Differences in the change in relative peak cue DA as a function of impulsivity. ***A***, Relative peak cue DA for the large reward cue (peak cue DA for the large reward cue minus peak cue DA for the small reward cue) decreased as a function of delay, but the decrease was weaker in impulsive rats. The raw data from which these difference scores were derived are: Low impulsivity, Large cue, 0 s: 103.52 ± 8.22 nM; small cue, 0 s: 61.42 ± 7.92 nM; large cue, 10 s: 57.57 ± 4.58 nM; small cue, 10 s: 45.36 ± 7.16 nM; large cue, 20 s: 31.35 ± 3.70 nM; small cue, 20 s: 32.31 ± 7.27 nM. High impulsivity, Large cue, 0 s: 74.85 ± 11.34 nM; small cue, 0 s: 52.29 ± 7.69 nM; large cue, 10 s: 44.32 ± 5.77 nM; small cue, 10 s: 43.01 ± 7.24 nM; large cue, 20 s: 37.20 ± 5.83 nM; small cue, 20 s: 30.13 ± 4.43 nM. Inset, The change in relative peak cue DA for the large reward cue from 0 to 20 s was smaller in impulsive rats, *p < 0.05. ***B***, Percentage choice of the large/delayed reward significantly correlated with the change in relative peak cue DA for the large reward cue from 0 to 20 s.

In addition to reduced cumulative DAergic release to cues, DA signaling in high impulsive rats had a blunted ability to vary as a function of increasing delay. First, we analyzed relative peak cue DA (peak DA for the large cue – peak DA for the small cue) across each delay. As Figure 5*A* shows, low impulsive rats exhibited a steeper shift in relative peak cue DA as a function of delay. Specifically, a two-way ANOVA showed a significant main effect of delay (*F*_(2,24)_ = 17.82, *p* < 0.001), no main effect of impulsivity group (*F*_(1,12)_ = 1.48, *p* = 0.248), but a significant interaction between delay and impulsivity group (*F*_(2,24)_ = 3.48, *p* = 0.047). To further understand this effect, we subtracted relative peak cue DA at no delay (0 s) from relative peak cue DA at long delay (20 s). As shown in Figure 5*A*, inset, we then compared this change in relative peak cue DA between low and high impulsive rats and found that high impulsive rats exhibited a significantly blunted drop in relative peak cue DA from the 0- to 20-s delay (*t*_(12)_ = 2.31, *p* = 0.039), suggesting that DA in these rats was less able to update the changing value of the cues. Finally, we ran a correlation between percentage choice of the large, delayed lever and the change in relative peak cue DA (relative peak cue DA at 20 s – relative peak cue DA at 0 s). As shown in Figure 5*B*, we found that a large change in relative peak cue DA significantly correlated with low impulsivity (*r* ^2^ = 0.42, *p* = 0.012); this finding was not significant when using data only from each individual delay (0 s alone, 10 s alone, and 20 s alone; *p* > 0.05). Critically, there were no interactions between impulsivity and drug history (all analysis of variance interactions, *p* > 0.05). Collectively, these results suggest that DA in high impulsive rats (independent of drug history) is dampened and is not modulated by delay or magnitude as robustly as it is in low impulsive rats.

**Figure 6.**
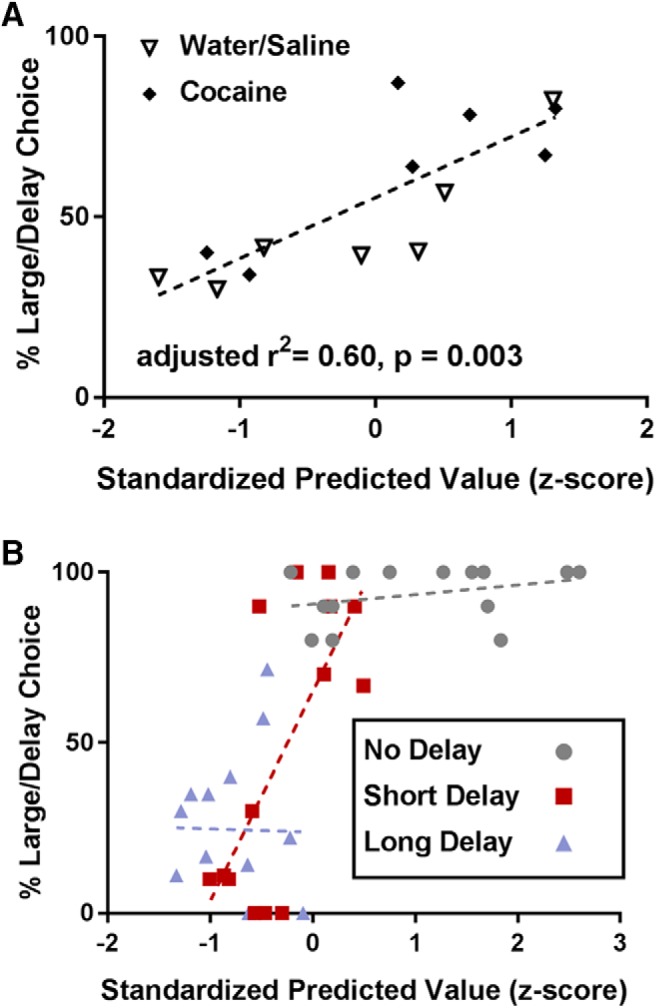
***A***, Cumulative cue DA and the change in relative peak cue DA have a very strong relationship with impulsivity when examined together using a linear regression model. Entering cumulative cue DA and the change in relative peak cue DA as independent factors into a multiple linear regression model significantly predicted impulsivity in the delay discounting task better than either factor did on its own. The *x*-axis is the standardized predicted value derived from the model. ***B***, Entering cumulative cue DA and relative peak cue DA as independent factors into a multiple linear regression model significantly predicted behavioral choice during the short delay (10 s) block, but not during the other blocks.

Although cumulative cue DA (Fig. 4) and the change in relative peak cue DA (Fig. 5) both predicted impulsive behavior, we were uncertain whether these were measuring two distinct DAergic processes or the same underlying DAergic mechanism. For example, rats with blunted overall DA may simply have less DA to be modulated by delay or magnitude; if so, cumulative cue DA and change in relative peak cue DA are likely measuring the same underlying mechanism and should therefore correlate with each other. However, we found that cumulative cue DA had no relationship to the change in relative peak cue DA (*r* ^2^ = 0.04, *p* = 0.495), suggesting that these were two distinct DAergic processes. Because these two independent events both predicted impulsive behavior, we also examined whether they predicted relatively nonoverlapping shares of the impulsivity variance using a linear regression model. There was a significant overall effect of the linear regression model (*F*_(2,11)_ = 10.74; adjusted *r* ^2^ = 0.60; *p* = 0.003; Fig. 6*A*), and both factors significantly predicted impulsivity (cumulative cue DA: β = 0.50, *t*_(11)_ = 2.80, *p* = 0.017; change in relative peak cue DA: β = −0.55, *t*_(11)_ = −3.06, *p* = 0.011), suggesting that these two factors did account for largely nonoverlapping shares of the impulsivity variance. Indeed, including both DAergic processes in the model predicted more variance (*r* ^2^ = 0.60, Fig. 6*A*) than did either alone [cumulative cue DA: *r* ^2^ = 0.37 (Fig. 4*C*), change in relative peak cue DA: *r* ^2^ = 0.42 (Fig. 5*B*)]. Importantly, when we looked at each delay independently (e.g. examining 0 s data only without any 10 s or 20 s data, etc.), we found significant effects for the 10-s delay data alone (linear regression model: *F*_(2,11)_ = 5.98; adjusted *r* ^2^ = 0.43; *p* = 0.017; cumulative cue DA: β = 0.57, *t*_(11)_ = 2.64, *p* = 0.023; relative peak cue DA: β = 0.62, *t*_(11)_ = 2.85, *p* = 0.016; Fig. 6*B*), but not the 0 s or 20 s data alone (all ps > 0.05). Thus, in addition to predicting behavior across all delays, the linear regression model also correlated with behavioral choice within the 10-s delay block, where high and low impulsivity rats’ choice behavior diverged the most (Fig. 4*A*).

## Discussion

Here, we investigated the role of a history of cocaine on rapid DA signaling dynamics during a delay discounting task. In controls, we found that DA release in the NAc core was modulated by both reward delay and magnitude, consistent with previous work (Saddoris et al., 2015). However, a history of cocaine self-administration had no effect on either delay discounting behavior or rapid DA release dynamics during the task. We next examined whether individual differences in impulsivity, independent of drug history, were related to DA release in the NAc core. We found that regardless of drug history, individual differences in impulsivity were related to DA release, as high impulsive animals exhibited dampened cue DA release that failed to robustly vary as a function of delay and magnitude. These findings are discussed in detail below.

### DA release dynamics are modulated by delay and magnitude but are unaltered by a history of cocaine self-administration

In controls, we demonstrated that rapid cue-induced DA release in the NAc core was modulated by differing delays and magnitudes, and that the amount of DA released predicted subsequent choice behavior. However, unlike our previous report (Saddoris et al., 2015), cue DA for the small reward cue was not larger than cue DA for the large reward cue at the longest (20 s) delay. Nevertheless, we also observed that greater relative peak cue DA release (change in peak DA) to the large cue during forced choice trials predicted greater percentage choice of the large reward as a function of delay during free choice trials. These findings compliment other literature demonstrating that DA activity in the NAc tracks the value of cues during decision-making behavior (Day et al., 2010; Gan et al., 2010; Sugam et al., 2012). Additionally, the present study confirms work in both animals and humans showing that activity in the ventral striatum is critically involved in evaluating reward during decision-making behaviors (McClure et al., 2004; Hariri et al., 2006; Roesch et al., 2007a).

We also found that a history of cocaine self-administration had no effect on either delay discounting behavior or rapid DA release in the NAc core. This was unexpected, given that a number of studies have shown that animals with a history of cocaine exhibit heightened delay discounting (Roesch et al., 2007b; Simon et al., 2007; Anker et al., 2009b; Mendez et al., 2010; Zuo et al., 2012; Hernandez et al., 2014; Mitchell et al., 2014). However, it is important to note that our lack of effect of cocaine history on delay discounting is not an isolated finding. Several other studies have reported either no effect of cocaine self-administration history on delay discounting (Broos et al., 2012), that cocaine history only impaired the subsequent ability of an acute injection of cocaine to alter delay discounting (but did not alter delay discounting itself; Winstanley et al., 2007), or that cocaine history only altered delay discounting when rats were tested in a cocaine-paired context (but not a neutral context; Xie et al., 2012). Collectively, these findings highlight the complexity of these phenomena. Interestingly, this complexity in cocaine’s actions may be unique to delay discounting, since impairments in NAc neural signaling and DA release dynamics during other forms of associative learning (e.g., second order conditioning, pavlovian to instrumental transfer, conditioned approach), have been reported using a similar self-administration regimen (Saddoris and Carelli, 2014, Saddoris et al., 2016a,b; Cameron et al., 2016).

### In impulsive rats, cue DA release is blunted and fails to robustly vary as a function of delay or magnitude

Although we found no effect of cocaine history on delay discounting behavior or DA release, we did find that individual differences in impulsivity were related to DA release in the NAc core, independent of drug history. That is, animals with high impulsivity had lower DA release during presentation of cues that signaled the upcoming reward. This effect was not modulated by delay or magnitude, suggesting that individuals with high impulsivity may have dampened cue DA regardless of the information signaled by the cue. Furthermore, this effect was restricted to cumulative cue DA in the 5-s period following the cue, suggesting that differences in impulsivity may partially reflect slower release dynamics following peak DA release. Our findings fit with other studies, where dampened DA release in the NAc following electrical stimulation is seen *in vitro* in rats classified as impulsive in a delay discounting task (Diergaarde et al., 2008), and high discounters have lower DA release in the NAc following systemic administration of amphetamine (Zeeb et al., 2016), although no differences in baseline DA are seen (Barbelivien et al., 2008; Zeeb et al., 2016). These findings, coupled with our own, suggest that events that trigger an increase in DA in the NAc (such as electrical stimulation, drugs, or cues) are less potent at triggering this release in impulsive rats. As such, this dampened DA signaling could render animals less able to distinguish between the relative values of cues, which may in part drive their heightened impulsivity.

In addition to the relationship between impulsivity and cue-evoked DA release, our study also demonstrates a second, more complex relationship with DA. Specifically, we found that the relative difference in peak cue DA release between the large and small cue failed to vary as a function of delay and magnitude in impulsive rats as robustly as it did in nonimpulsive rats. This may suggest that an inability to track the changing values of the cues is partially responsible for impulsive behavior, although further research would be needed to test this possibility. Importantly, a number of studies show that poor timing ability is associated with more impulsive delay discounting in rats (Marshall et al., 2014; McClure et al., 2014). This inability to time events may therefore impair encoding of cue value as a function of delay (similar to that seen in the current study), which could subsequently lead to more impulsive behavior. Alternatively, high impulsive rats may be using a different task strategy that does not require cue value discrimination, such as habitually choosing the immediate lever as soon as the delayed trials begin.

Additionally, we found that cumulative cue DA and the change in relative peak cue DA were uncorrelated with each other and both independently predicted impulsive behavior in the delay discounting task. Indeed, including both in a linear regression model predicted impulsive behavior better (accounting for 60% of the variance) than either variable did alone (cumulative cue DA accounting for 37% of the variance, change in relative peak cue DA accounting for 42% of the variance). Furthermore, these two processes together predicted choice behavior within the short delay block (10 s), which is where the high and low impulsive rats diverged the most in their choice behavior. Thus, our data suggest that there may be two discrete processes at the level of DA in the NAc core that underlie delay discounting behavior. As neither of these DAergic processes correlated with errors, omissions, or response latency, future work will be needed to establish whether any particular behaviors (e.g. cue value discrimination, timing, motivation) are uniquely associated with one or the other process.

The role of NAc core DA in the individual differences seen in delay discounting is not well understood, but recent studies have provided some insight. In human subjects, high impulsivity in the delay discounting task correlates with task-evoked hyperactivity in the ventral striatum (Hariri et al., 2006), blunted striatal DA release in response to a gambling task (Joutsa et al., 2015), and decreased striatal D2/D3 receptor density (Ballard et al., 2015; Joutsa et al., 2015), although another study found no relationship between discounting and ventral striatal DA synthesis (Smith et al., 2016). Critically, it is important to note that within the rodent literature there are no relationships seen between individual differences in delay discounting and DA receptor or transporter expression in the NAc core (Loos et al., 2010; Simon et al., 2013). Thus, the important difference at the level of the NAc core may be through phasic DA release per se (as seen in the work of Diergaarde et al., 2008; Zeeb et al., 2016; and the current study) rather than DA receptor function/availability or tonic DA release. From a more causal perspective, baseline levels of impulsivity can impact the effects of systemic amphetamine (which indirectly increases DA) on delay discounting (Barbelivien et al., 2008), as well as the effects of manipulations of the lateral orbitofrontal cortex (Zeeb et al., 2010), anterior insula (Pattij et al., 2014), and NAc core (Moschak and Mitchell, 2014) on delay discounting. The latter study found that inactivation of the NAc core altered delay discounting in low impulsivity rats, but had no effect on high impulsivity rats. This result, coupled with the current finding that high impulsivity rats have dampened NAc core DA that fails to robustly vary as a function of delay or magnitude, may suggest that the NAc core and its accompanying DA fail to fully engage the decision-making process in high impulsivity rats. Additionally, the orbitofrontal cortex and insula listed above are also innervated by DA (Berger et al., 1991; Jasmin et al., 2004), and may involve similar relationships between DA release and impulsivity.

### Concluding comments

In summary, we show that cue-induced DA in the NAc core is modulated by delay and magnitude during a delay discounting task. Critically, high impulsivity rats exhibited dampened cue DA release which failed to robustly vary as a function of delay and magnitude, independent of a history of cocaine. These findings augment a growing literature demonstrating the impact of individual differences in delay discounting and other forms of impulsivity on behavior and neural function (Dalley et al., 2007; Diergaarde et al., 2008; Loos et al., 2010; Simon et al., 2013; Zeeb et al., 2016; Moschak et al., 2017). Future studies should further investigate the differences in neurocircuitry between high and low impulsive individuals.

## Synthesis

Reviewing Editor: Karen Szumlinski, University of California at Santa Barbara Decisions are customarily a result of the Reviewing Editor and the peer reviewers coming together and discussing their recommendations until a consensus is reached. When revisions are invited, a fact-based synthesis statement explaining their decision and outlining what is needed to prepare a revision will be listed below. Note: If this manuscript was transferred from JNeurosci and a decision was made to accept the manuscript without peer review, a brief statement to this effect will instead be what is listed below.

I have read the authors' rebuttal in response to their prior review of their article and found the revised report much improved. I particularly appreciated the authors' efforts to re-analyze the data as suggested and to revise the discussion. As such, I opted to defer from another round of external review and I am happy to notify the authors that I have decided to accept the manuscript as is.
